# Prevalence of rotavirus and adenovirus associated with diarrhea among displaced communities in Khartoum, Sudan

**DOI:** 10.1186/1471-2334-13-209

**Published:** 2013-05-08

**Authors:** Wafa I Elhag, Humodi A Saeed, El Fadhil E Omer, Abdelwahid S Ali

**Affiliations:** 1 Department of Microbiology, Faculty of Medical Laboratory Sciences, Al Neelain University, Khartoum, Sudan; 2 Department of Microbiology, College of Medical Laboratory Sciences, Sudan University of Science and Technology, Khartoum, Sudan; 3 Faculty of Medicine, King Khalid University, Riyadh, Saudi Arabia

**Keywords:** Diarrhea, Rotavirus, Adenovirus, PCR, Displaced camps, Khartoum

## Abstract

**Background:**

Diarrheal diseases represent a major worldwide public health problem particularly in developing countries. Each year, at least four million children under five years of age die from diarrhea. Rotavirus, enteric adenovirus and some bacterial species are the most common identified infectious agents responsible for diarrhea in young children worldwide. This study was conducted to determine prevalence of rotavirus and adenovirus associated with diarrhea among displaced communities in Khartoum state, Sudan.

**Methods:**

A total of seven hundred and ten patients, children and adults, suffering from diarrhea were examined. The clinical history, socio-demographic characteristics, physical examination findings and laboratory investigations were recorded. Stool samples or rectal swabs were collected and tested for rotavirus and adenovirus antigens using the immuno-chromatography test (ICT). Characterization of the identified Rotaviruses, as a major cause of diarrhea, was then made using real time-reverse transcription PCR. To make the study legal, an ethical clearance was obtained from Sudan Ministry of health- Research Ethical Committee. Written consent was taken from adult subjects, and also from children mothers.

The participants were informed using simple language about the infection, aim of the research and the benefits of the study.

**Results:**

Out of the 710 patients, viral pathogens were detected in only 99 cases (13.9%). Of the 99 cases of viral diarrhea, 83 (83.8%) were due to rotaviruses while 16 (16.2%) attributed to adenovirus. Of the 83 rotaviruses identified, 42 were characterized by RT-PCR, of these 40 (95.2%) were proved as type A (VP6), and 2 (4.8%) type C (VP7). Type C (VP7) rotavirus was detected in samples collected from children under 5years only.

**Conclusions:**

In conclusion, most cases of viral diarrhea are found to be caused by rotavirus especially among children less than five years. Most of the identified rotavirus belonged to type A (VP6).

It was also evident that most patients are those who drank untreated water obtained from donkey carts source and who had no access to latrines, and lived in poor environmental conditions would acquire diarrheal infection.

## Background

Diarrhea remains the second leading cause of death around the world for children under 5 years of age [1].

Rotavirus is the most common cause of severe diarrhea in children, resulting in the hospitalization of approximately 55,000 children each year in the United States [2,3]. In the developing world rotavirus may account for 1 million childhood deaths as well as significant morbidity each year [4].

Rotavirus has also been implicated as an etiological agent of diarrhea in older children, adult human, young and adult animals, including calves and piglets. Among rotaviruses are the agents of human infantile diarrhea, Nebraska calf diarrhea, and epizootic diarrhea in infant mice [5].

Rotavirus is endemic worldwide; the infection is associated with high rates of morbidity throughout the world and high rates of mortality in developing countries. In the United States, rotavirus infection is responsible for approximately 3 million cases of diarrhea in children less than 5 years old each year, although these infections cause relatively few deaths in United States. However, in developing countries, rotavirus gastroenteritis account for more than 800,000 childhood deaths per year due to poor nutrition and health care [6]. Children in the poorest countries account for 82% of rotavirus deaths [7].

The main symptoms of rotavirus gastroenteritis (RVGE) are fever, abdominal pain, lethargy, diarrhea and vomiting that may lead to hypovolemic shock and dehydration [8,9]. Severe cases may lead to death [10].

There are three rotavirus groups, A, B and C, which are both antigenically and genetically distinct. The inner capsid protein, VP6 is the common or group-specific antigen. Group A rotaviruses are the predominant cause of rotavirus disease throughout the world. More than 95% of all children have been infected by group A rotaviruses by the age of 4 and in developing countries infection occurs primarily between 4 months and 2 years of age [11].

Displaced persons around Khartoum state live in poor, un healthy condition in camps, suffering from diarrhea, the diarrhea etiology remain obscure (in particular, rotavirus prevalence remains largely unknown due to the absence of commercially available diagnostic tests) which is very important in deciding treatment, prevention, and control of diarrhea. For all these reasons this sudy aimed to know the viral etiology associated with diarrhea in displaced camps.

## Methods

### Type and duration of the study

A descriptive cross-sectional study aiming at determination of viral etiology of diarrhea among displaced persons in Khartoum state, Sudan was conducted. The study was carried out during April 2006–September 2008, at Mandella and Wad Elbashir displaced camps health centers in Khartoum State.

### Samples

A total number of 710 stool specimens were collected from patients with diarrhea, using sterile clean containers or rectal swabs.

**Figure 1:**
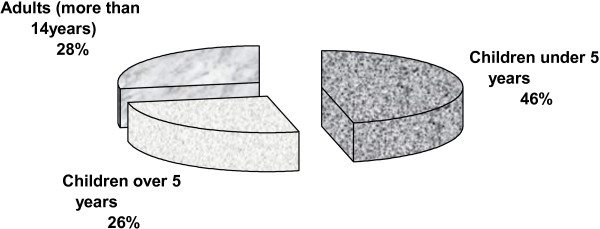
Age distribution of study group.

Displaced persons, who were residents of the camp and had diarrhea complaints, were targeted in this study. Others who did not live in camps, and who have no diarrhea were excluded. The patients studied covered adults and children. Most of patients (46%) were children under 5 years (Figure 1).

Ethical approval was obtained from Research Ethical Committee- Sudan Ministry of Health. Written consent was taken from adult subjects, and also from children mothers.

The participants were informed using simple language about the infection, aim of the research and the benefits of the study.

### Immunochromatographic test (ICT)

The stool specimens were tested as soon as possible after collection, and were directly tested with Immunochromatographic test (ICT) (Coris Bioconcept, ltd- Belgium) for rotavirus and adenovirus. This is a ready-to-use test based on the use of a homogenous membrane system with colloidal gold particles. The fecal sample was diluted in the dilution buffer supplied with the test. A nitrocellulose membrane is sensitized with antibodies directed against rotavirus and adenovirus (test lines). The test was carried out as described by Weitzel *et al.* (2007) [12].

### Reverse transcription real time PCR

Specimens found positive for rotavirus, selected from different age groups were characterized using 3 sets of primers, specific for rotavirus type A, and C using reverse transcription real time PCR. The RNA was extracted directly from stool specimens with viral RNA isolation kit NucleoSpin® RNA Virus (Genekamp-ltd), using protocol of viral RNA isolation from cell free biological fluids according to Villahermosa *et al.* (2000) [13]. The extracted RNA was preserved at – 80°C for one week before starting PCR experiments.

Real time- Reverse Transcription PCR was performed using the SensiMix One-Step Kit (Quantace- ltd), SYBR® Green 1 protocol; SYBR Green 1 binds to double-stranded DNA and emits light when excited.

Components of master mix used for reaction were 50xconc SYBR® Green 1, SensiMix One-Step (reaction buffer, reverse transcriptase, heat activated DNA Polymerase, dNTPs, 6mM MgCl2, internal reference, stablizers), MgCl2, RNase inhibitor, dH2O (Quantace, ltd), Forward Primer (5 μM), and Reverse Primer (5 μM) (eurofins MWG GmbH- ltd).

Specific primers for target RNA (Rotavirus A, and C) designed from National MWG GmbH- ltd.

Rotavirus A viral protein 6 (VP6) two primers, and Rotavirus C (VP7) one primer as target genes, the primers sequence were:

RotaA- fwd1 5, -GGA TGT CCT GTA CTC CTT GTC AAAA −3,

RotaA- rev1 5, -TCC AGT TTG GAA CTC ATT TCC −3,

RotaA- fwd2 5, - GGA GGT TCT GTA CTC ATT GTC AAA AA −3,

RotaA- rev2 5, -TCC AGT TTG AAA GTC ATT TCC ATT −3,

RotaC- fwd 5, -TTA GAT ACT ACA AGT AAT GGA ATC GGA TGT −3,

RotaC- rev 5, -TGG GTG TCA TTT GAT ACA ACT TCA −3,

Every specimen was tested with the three different primers in duplicate. Twenty μl from PCR Master Mix and 5 μl of RNA template were added separately. Twenty μl from PCR Master Mix and 5 μl dH2O was also added in two well of the plate in a duplicate form, as non template control (NTC). Then the plate was sealed using thin plastic film, and heat sealer (Combi Ltd) before insertion into Real-time PCR machine (Quantica, Techne Ltd). The plate was run in Quantica thermo cycler using Quansoft software (Quantica, Techne- ltd) according to the following program; Reverse Transcription step (one cycle), 42°C 30 min.; Enzyme activation (one cycle), 95°C 10 min.; Amplification (35 cycles), 95°C for 15 seconds; (denaturation); 58°C for 30 seconds (annealing); 72°C for 60 seconds (extension).

PCR products were then analyzed using (Plus/minus scoring methods) which used to record the presence or absence of a PCR product (Qualitative analysis). A non template control (NTC) was used to set confidence thresholds above which all unknowns were scored positive and negative. Samples between the two threshold values were scored as undetermined.

Data was analyzed by Statistical package of social sciences (SPSS) programmed for windows, version 11.5.

## Results

**Figure 2:**
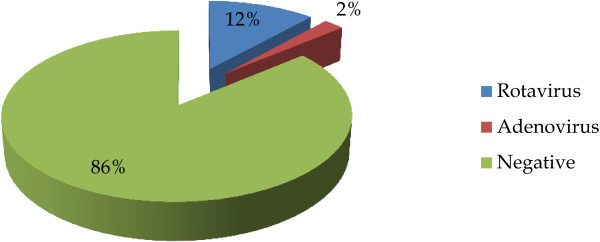
Frequency of rotaviruses and adenoviruses among the study group (n = 710) using Immuno-chromatography test (ICT).

**Figure 3:**
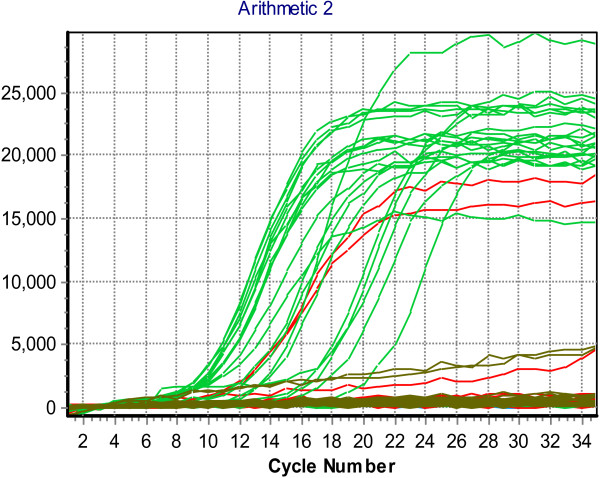
A real sigmoid curves of specimens in plate No (1) (including positive and negative results).

**Figure 4:**
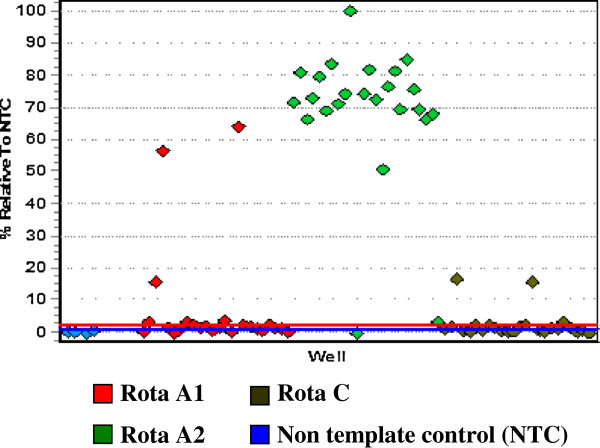
Plus/Minus scoring analysis showing positive samples (plate 1), above base line (threshold).

**Figure 5:**
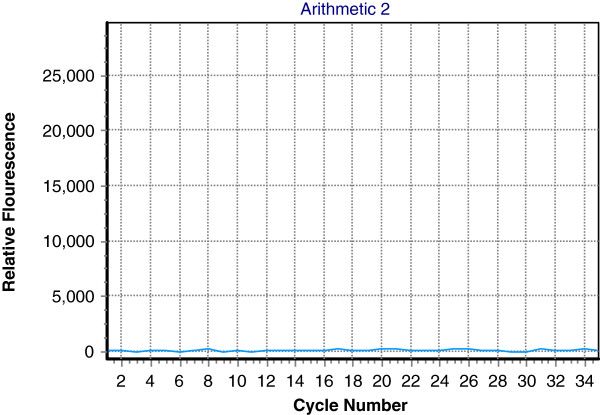
The reading of non template control (plate 1).

**Figure 6:**
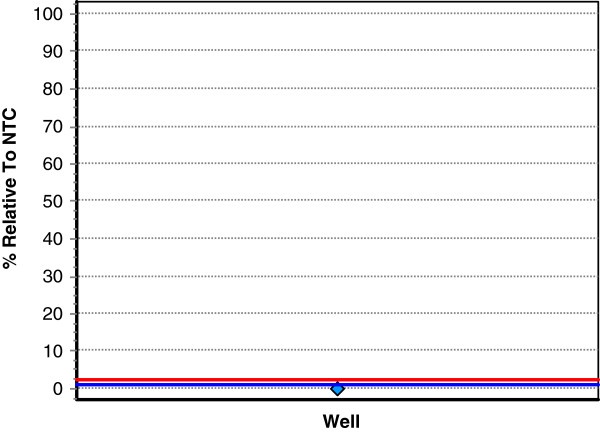
Plus/Minus scoring analysis of non template control (NTC) plate (1).

Among the total of 710 stool specimens, viral pathogens were identified in 99 (13.9%) of patients with diarrhea. Out of the 99 cases of viral diarrhea, 83 (83.8%) were due to rotaviruses (VP6), and 16 (16.2%) to adenovirus (Figure 2). Most of viral enteropathogens were detected among children less than 5 years of age (Table 1). Of the 83 rotavirus strains, 42 were characterized by molecular technique, using real time reverse- transcription PCR. Out of these 40 (95.2%) were proved as type A (VP6), and 2 (4.8%) type C (VP7). However, all type C rotavirus was detected among children less than five years. Figures 3, 4, 5 and 6 show the results of one real time RT-PCR experiment.

**Table 1:** Distribution of viral pathogens according to age

**Viral pathogens**	**5 years n = 330 (100%)**	**Children 5 years n = 182 (100%)**	**Adults n = 198 (100%)**	**Total n = 710 (100%)**
**Rotavirus**	53 (16.1%)	12 (6.6%)	18 (9.1%)	83 (11.7%)
**Adenovirus**	9 (2.7%)	1 (0.5%)	6 (3.1%)	16 (2.2%)
**Total**	62 (18.7%)	13 (7.1%)	24 (12.1%)	99 (13.9%)

As for the sociodemographic factors associated with diarrhea in this study, most of patients were found depending on donkey carts as water source 390 (54.9%), followed by 200 (28.8%) patients getting their water directly from water pump, and 120 (16.9%) patients are using storage tanks as a water supply. 260 (36.6%) of the patients are drinking untreated water, At the same time 66.9% of patients under study had no disposal latrines. Household status was also considered, and found most of the patients suffering from crowdness, about 80% of them live in shelters containing more than five residents.

## Discussion and conclusion

Diarrhea are major causes of morbidity, with attack rates ranging from 2 to 12 or more illnesses per person per year in developed and developing countries. In addition, diarrheal illnesses account for an estimated 12,600 deaths each day in children in Asia, Africa, and Latin America. The causes of diarrhea include a wide range of viruses, bacteria, and parasites, many of which have been recognized only in the last decade [14].

More than 30 million refugees and internally displaced persons in developing countries are currently dependent on international relief assistance for survival. Displaced populations in northern Ethiopia (1985) and southern Sudan (1988) have suffered the highest mortality rates. Although mortality rates have risen in all age groups, the greatest have been recorded in children 1–14 years old. The major causes of death have been measles, diarrhea, acute respiratory tract infections and malaria [15].

In the rehabilitation camps in Khartoum State (Mandella & Wad Elbashir) were holding more than 110,000 Sudanese displaced. The poor nutrition, overcrowding, lack of water, and inadequate sanitation made this population especially vulnerable to diarrhea.

In this study viral pathogens were incriminated in 13.9% of patients with diarrhea. Many of them were attributed to rotaviruses while some adenovirus. Regarding age distribution, rotavirus was the main viral etiologic agent of diarrhea among children under five years with 16.1% frequency rate. This was almost comparable to results of Mertens, *et al.* (1990), who study diarrhea on childhood in 1987 in Sri Lanka [16], and slightly lower than Youssef, *et al.* (2000) results on etiology of acute diarrhea in Jordanian children less than five years of age [17].

The present findings were also similar to that reported by Adkins and co workers who found that rotavirus infection occurred in both children and adults throughout the year and was the most frequently identified cause of diarrhea in children under five years of age Manila in 1983 and 1984 [18].

The prevalence of rotavirus as the main cause of diarrhea in refugee camp was also shown by Nimri, and Hijazi (1996) in children in a refugee camp in Jordan, concluded that rotavirus antigens were detected with 35% frequency rate [19]. Also another study conducted by Georges and his colleges who concluded that rotavirus was the most frequently identified pathogen among children less than 18 months old in Central African Republic [20].

In several international reports, it was evident that viral pathogens are the most common cause of gastroenteritis in developed countries Worldwide [16,21-23]. Gastrointestinal rotavirus infections result in an estimated 440,000 deaths in children fewer than five years of age [24]. Adenoviruses are second only to rotavirus as the most important causative agents of acute infantile gastroenteritis [25]. The present results established that rotaviruses and adenoviruses play a major role in diarrhea among displaced communities especially less than 5 year’s children.

Rotaviruses detected in this study (from different age group) were further characterized using the real time PCR technique. The results obtained revealed that 95.2% were type A, and 4.8% type C. Type C rotavirus was detected among children less than 5 years only. Group A rotaviruses, as predominant and result in severe diarrheal diseases in infants and young children, was also reported by Urbina *et al.* (2003) in Colombia [26], and in a relief camp in India [27].

Several authors reported that group C rotaviruses, is an emerging cause of gastroenteritis in children over 2 years old and in adults, in both sporadic cases and outbreaks worldwide [28-32].

It was also reported that various molecular techniques have been exploited for the development of highly sensitive and rapid assays for the detection of causative agents of viral gastroenteritis [33-35]. Reverse transcription-PCR has been reported as a test for increasing the detection rate of rotavirus A by up to 48% compared to EIA or electron microscopy [36,37].

As a conclusion, this study revealed that 13.9% of patients studied were infected by viral enteropathogens. Children under 5 years were mostly infected, and the major viral pathogen causing diarrhea in displaced camps in Khartoum was rotavirus, the most commonly rotavirus being type A (95.2%).

The study emphasizes the need for continuous monitoring of viral enteropathogens for successful treatment and control of diarrhea, and also for the development of public health policy for populations in displaced camps.

## Competing interests

The authors declare that they have no competing of interests.

## Authors’ contributions

WIE, designed and conducted the study and drafted the paper. HAS, designed the laboratory procedures, and contributed in drafting the paper. EFEO, coordinated the laboratory quality control^,^ revised the manuscript critically for important intellectual content. ASA, Managed the data and performed the statistical analyses. All authors read and approved the final manuscript.

## Pre-publication history

The pre-publication history for this paper can be accessed here:

http://www.biomedcentral.com/1471-2334/13/209/prepub
